# High-Tech Acupuncture and Integrative Laser Medicine 2013

**DOI:** 10.1155/2013/590718

**Published:** 2013-12-02

**Authors:** Gerhard Litscher, Xin-Yan Gao, Lu Wang, Bing Zhu

**Affiliations:** ^1^Stronach Research Unit for Complementary and Integrative Laser Medicine, Research Unit of Biomedical Engineering in Anesthesia and Intensive Care Medicine, TCM Research Center Graz, Medical University of Graz, Auenbruggerplatz 29, 8036 Graz, Austria; ^2^Institute of Acupuncture and Moxibustion, China Academy of Chinese Medical Sciences, Beijing 100700, China

This is an annual special issue. The current issue is the 2013 issue which includes eleven interesting papers.

Acupuncture has been used for medical treatment for thousands of years. A large number of empirical data is available, but the technical quantification of effects was not possible up to now. Using electroacupuncture, needle, or laser stimulation and modern biomedical techniques, it was possible for the first time to quantify changes in biological activities caused by acupuncture.

As mentioned above, this special issue contains eleven publications, of which six are related to laser acupuncture, three to electroacupuncture, two to needle acupuncture, and one to photoluminescent bioceramic material. Apart from body acupuncture, special emphasis is also given to auricular acupuncture. One review article, 22 pages in length, deals with the topic auricular acupuncture with laser. The investigations cover animal experimental studies and studies in healthy volunteers, preterm neonates, and patients, as well as basic and clinical research on evidence-based high-tech acupuncture, integrative laser irradiation, and translational medicine. 

It has to be mentioned that this annual special issue 2013 contains, among others, the following topics:

- modernization of acupuncture (evidence-based medicine, integrative laser medicine),
- high-tech acupuncture,
- development of innovative acupuncture stimulation methods,
- scientific evaluation of complementary medical methods (acupuncture, electroacupuncture, and low level laser therapy),
- clinical effects of acupuncture,
- intravenous laser blood irradiation,
- laser needle acupuncture,
- red and infrared laser stimulation,
- auricular acupuncture with laser,
- biomedical assessment of acupuncture,

Modernization of acupuncture is performed by high-tech acupuncture and integrative laser stimulation. Eastern and Western medicine are getting closer to each other using modern biomedical engineering technology, as described in this annual special issue. 

